# Plk1-Dependent Recruitment of γ-Tubulin Complexes to Mitotic Centrosomes Involves Multiple PCM Components

**DOI:** 10.1371/journal.pone.0005976

**Published:** 2009-06-19

**Authors:** Laurence Haren, Tim Stearns, Jens Lüders

**Affiliations:** 1 Institut de Sciences et Technologies du Médicament de Toulouse, Centre National de la Recherche Scientifique/Pierre Fabre, Toulouse, France; 2 Department of Biology, Stanford University and Department of Genetics, Stanford University Medical School, Stanford, California, United States of America; 3 Cell & Developmental Biology Programme, Institute for Research in Biomedicine (IRB), Barcelona, Spain; University of Birmingham, United Kingdom

## Abstract

The nucleation of microtubules requires protein complexes containing γ-tubulin, which are present in the cytoplasm and associate with the centrosome and with the mitotic spindle. We have previously shown that these interactions require the γ-tubulin targeting factor GCP-WD/NEDD1, which has an essential role in spindle formation. The recruitment of additional γ-tubulin to the centrosomes occurs during centrosome maturation at the G2/M transition and is regulated by the mitotic kinase Plk1. However, the molecular details of this important pathway are unknown and a Plk1 substrate that controls γ-tubulin recruitment has not been identified. Here we show that Plk1 associates with GCP-WD in mitosis and Plk1 activity contributes to phosphorylation of GCP-WD. Plk1 depletion or inhibition prevents accumulation of GCP-WD at mitotic centrosomes, but GCP-WD mutants that are defective in Plk1-binding and -phosphorylation still accumulate at mitotic centrosomes and recruit γ-tubulin. Moreover, Plk1 also controls the recruitment of other PCM proteins implicated in centrosomal γ-tubulin attachment (Cep192/hSPD2, pericentrin, Cep215/Cdk5Rap2). Our results support a model in which Plk1-dependent recruitment of γ-tubulin to mitotic centrosomes is regulated upstream of GCP-WD, involves multiple PCM proteins and therefore potentially multiple Plk1 substrates.

## Introduction

In mitosis centrosomal and non-centrosomal microtubule nucleation pathways contribute to the formation of the bipolar spindle [1]–[4]. In late G2 cells prepare for mitosis and increase size and microtubule nucleating activity of the duplicated centrosomes. This is accomplished by the recruitment of additional pericentriolar material (PCM) to the centrosomes including proteins involved in microtubule nucleation and organization, such as γ-tubulin [5]. This process, also termed centrosome maturation, is critical for the function of centrosomes as microtubule organizing centers in mitosis, and depends on the activity of mitotic kinases such as Polo-like kinase 1 (Plk1) [6]. Interfering with Plk1 function by RNAi or specific inhibitors prevents the recruitment of γ-tubulin to mitotic centrosomes and inhibits the centrosomal microtubule nucleation pathway. Moreover, similar to γ-tubulin depletion or mislocalization, suppression of Plk1 activity causes loss of centrosome separation and formation of monopolar spindles [7]–[12]. To date, a Plk1 substrate that controls γ-tubulin recruitment in a phosphorylation-dependent manner has not been identified.

The γ-tubulin ring complex (γTuRC) is a large, multisubunit protein complex consisting of multiple copies of γ-tubulin and at least 6 additional proteins [1]–[3]. Several centrosome proteins have been suggested to attach the γTuRC to the PCM of the centrosome including the recently identified γTuRC component GCP-WD/NEDD1 [13], [14]. GCP-WD is specifically required for the localization of γ-tubulin to centrosomes in interphase and mitosis, but not for the localization of other PCM proteins. It behaves like a true γTuRC subunit but does not require the γTuRC for localization to the centrosome. Its unique properties suggest that it is the attachment factor most proximal to the γTuRC. In addition to centrosomal attachment GCP-WD mediates the interaction of the γTuRC with the mitotic spindle [13], [14]. Spindle localization of γTuRCs requires phosphorylation of GCP-WD at serine 418 and contributes to proper spindle assembly, possibly by nucleation of additional microtubules within the spindle [13]. Mutation of serine 418 to alanine abolishes spindle localization of GCP-WD and of γ-tubulin without affecting their accumulation at mitotic centrosomes. GCP-WD phosphorylation promotes interaction with the augmin complex, which was recently shown to be required for the spindle localization of the γTuRC [15]–[18]. It is not known whether centrosome localization of GCP-WD in mitosis is also controlled by phosphorylation.

As a γ-tubulin targeting factor and a mitotic phosphoprotein GCP-WD might be the key to understanding Plk1-dependent recruitment of γ-tubulin to mitotic centrosomes. We used depletion of Plk1 by RNAi and a recently developed Plk1 inhibitor to investigate a potential role of GCP-WD in this process.

## Results

### Plk1 regulates the amount of GCP-WD at centrosomes and spindle microtubules

**Figure 1:**
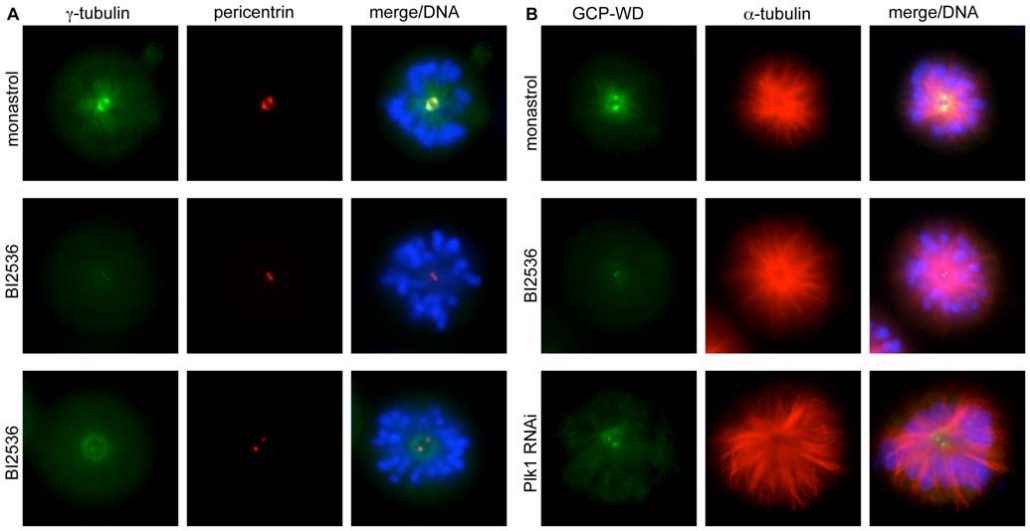
Plk1 activity is required for localization of γ-tubulin and GCP-WD to centrosomes and spindle microtubules. (A) HeLa cells treated with monastrol or BI2536 were fixed and immunostained for γ-tubulin and pericentrin.DAPI was used to label DNA. (B) HeLa cells treated with monastrol, BI2536 or siRNA against Plk1 were fixed and immunostained for GCP-WD and α-tubulin. DNA was visualised using DAPI.

To test how Plk1 controls the recruitment of γ-tubulin to mitotic centrosomes we incubated HeLa cells with the recently described Plk1 inhibitor BI2536 [12], [19] or depleted Plk1 by RNAi. Both treatments resulted in the formation of monopolar spindles and a prometaphase arrest, as described [8], [12] (Fig. 1A and 1B). Staining with pericentrin-specific antibodies was relatively weak in such cells, but revealed the presence of two centrosomes at each monopole, whereas centrosomal γ-tubulin was barely detectable [8], [12] (>90% reduction, Fig. 1A). In contrast, treatment of cells with monastrol, which induces monopolar spindles and prometaphase arrest by inhibiting the kinesin Eg5 [20], had no effect on γ-tubulin-recruitment to centrosomes (Fig. 1A). In addition to centrosomes, γ-tubulin also localizes to the region of kinetochore microtubules in the mitotic spindle [5], [21]. In monopolar spindles produced by treatment with monastrol this results in a pattern of γ-tubulin staining that surrounds the monopole in a radial fashion (Fig. 1A). A similar staining pattern can be observed in rare monopolar spindles in untreated cells (data not shown). Plk1 inhibition also impaired this localization of γ-tubulin to spindle microtubules. Spindle-associated γ-tubulin was often undetectable or, in cells with more γ-tubulin remaining at the centrosome, was confined to a region close to the spindle monopole (Fig. 1A, bottom). We tested whether γ-tubulin mislocalization after Plk1 inhibition resulted from the loss of the targeting factor GCP-WD from centrosomes and spindle microtubules by probing with GCP-WD-specific antibodies. Indeed, GCP-WD localization to these structures was strongly reduced in cells treated with the Plk1 inhibitor or depleted of Plk1 compared to monastrol-treated cells (>90% reduction, Fig. 1B). As γ-tubulin recruitment to mitotic centrosomes strictly depends on GCP-WD [13], [14], a possible mechanism would be that Plk1 controls mitotic γ-tubulin recruitment via GCP-WD phosphorylation.

### Plk1 activity contributes to GCP-WD phosphorylation in mitosis

**Figure 2:**
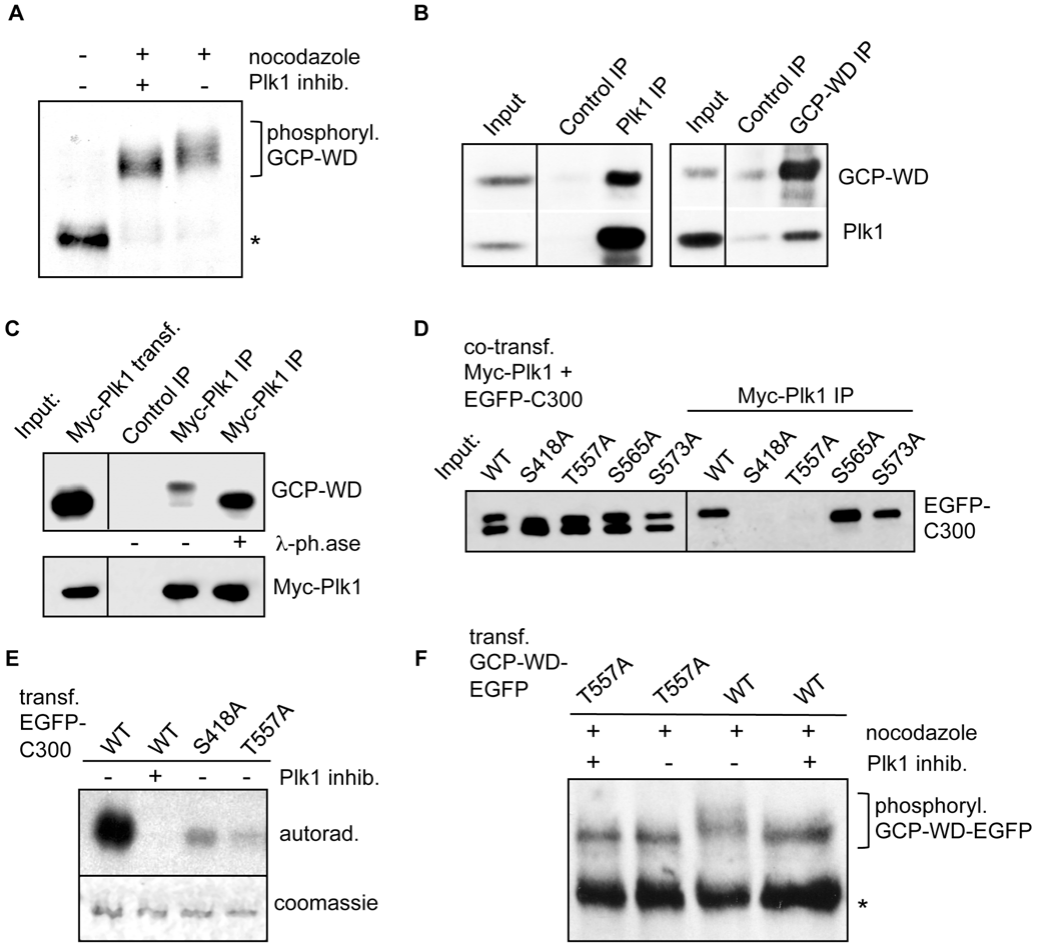
Plk1 interacts with GCP-WD in mitosis and contributes to its mitotic phosphorylation. (A) Hela cell lysates immunoblotted for GCP-WD. Cells were synchronised in mitosis by nocodazole treatment. BI2536 was added for 90 min before harvesting the cells by shake-off. The asterisk indicates the position of unphosphorylated GCP-WD. (B) Coimmunoprecipitation of endogenous GCP-WD and Plk1. Proteins were immunoprecipitated from mitotic lysates obtained from nocodazole-arrested HeLa cells after shake-off with antibodies against HA (Control IP), Plk1 and GCP-WD, as indicated. Precipitates were separated by gel electrophoresis, blotted and probed with anti-GCP-WD and anti-Plk1 antibodies. (C) Myc-tagged Plk1 was immunoprecipitated from extracts prepared from asynchronously growing HeLa cells after transient transfection. Unspecific mouse IgG was used as a control (Control IP). Precipitates were treated either with buffer alone or with λ-phosphatase and analyzed by westernblotting with anti-Myc and anti-GCP-WD antibodies. (D) After cotransfection of Myc-tagged Plk1 with C-terminal EGFP-tagged wildtype or mutant fragments of GCP-WD (EGFP-C300) in HeLa cells, Myc-tagged Plk1 was immunoprecipitated from non-synchronous cultures and precipitates were immunoblotted using anti-GFP antibody. (E) EGFP-tagged C-terminal GCP-WD wt or mutant fragments were transiently expressed in HeLa cells and immunoprecipitated after nocodazole treatment. Precipitates were subjected to an *in vitro* phosphorylation assay in the absence or presence of BI2536 to inhibit Plk1 activity. After SDS-PAGE the immunoprecipitated proteins were detected by coomassie staining, incorporation of [γ-32P] was detected by autoradiography. (F) Lysates from U2OS cells expressing EGFP-tagged GCP-WD wt or T557A mutant were immunoblotted for GCP-WD. Cells were synchronized in mitosis by nocodazole treatment. BI2536 was added for 90 min before harvesting the cells by shake-off. The asterisk indicates the position of unphosphorylated GCP-WD-EGFP.

GCP-WD is phosphorylated in mitosis, resulting in a mobility shift in SDS-PAGE [13], [14]. Treatment of mitotic cells with a Cdk inhibitor reverses this mobility shift, as does mutation of serine 418, a consensus Cdk1 phosphorylation site, to alanine, suggesting that GCP-WD phosphorylation is mainly driven by the mitotic kinase Cdk1 [13]. We tested whether Plk1 inhibition also affects the phosphorylation of GCP-WD. Mitotic cells arrested in prometaphase where obtained by shake-off after treatment with nocodazole in the absence or presence of the Plk1 inhibitor BI2536. Western blotting using high resolution SDS-PAGE and probing for GCP-WD revealed that inhibition of Plk1 slightly reduced the mobility of GCP-WD (Fig. 2A).

### Plk1 interacts with GCP-WD in a phosphorylation-dependent manner

Immunoprecipitation of endogenous Plk1 or GCP-WD from nocodazole-arrested HeLa cells and probing with anti-GCP-WD or anti-Plk1 antibody, respectively, demonstrated that Plk1 interacts with GCP-WD in mitosis (Fig. 2B). GCP-WD also interacted with transiently expressed myc-tagged Plk1 immunoprecipitated from asynchronously growing cells. In this case the phosphorylated form of GCP-WD preferentially coprecipitated with the kinase as revealed by phosphatase treatment after immunoprecipitation (Fig. 2C). We identified the Plk1-binding region in GCP-WD by coexpression of Myc-tagged Plk1 with EGFP-tagged GCP-WD fragments followed by immunoprecipitation. In this assay Myc-Plk1 did not interact with the WD40 repeat-containing N-terminal half of GCP-WD (data not shown). The C-terminal half of GCP-WD (C300) was sufficient for the interaction with Plk1 (Fig. 2D). As for endogenous GCP-WD (Fig. 2C), the interaction occurred with a phosphorylated form of fragment C300 (Fig. 2D) and phosphatase treatment prior to immunoprecipitation prevented coprecipitation of Plk1 and the C300 fragment (data not shown).

The efficient recognition and phosphorylation of Plk1 substrates requires binding of the polo box domain of the kinase to phosphopeptide motifs (consensus S-pS/pT-P) on the surface of the substrates [6], [22], [23]. Remarkably, the S418A mutation abolished interaction with Plk1 (Fig. 2D). Serine 418 and adjacent residues do not match the consensus of a polo box docking site, however phosphorylation of serine 418 might trigger subsequent phosphorylation of a polo box binding site elsewhere in the molecule, which would prime it for Plk1 binding. Consistent with this model, deletion of the last 121 amino acids (aa 546–667) from the C-terminal half of GCP-WD, which does not include serine 418, also abolished Plk1 binding (data not shown). We tested whether a consensus polo box binding motif surrounding threonine 557 is involved in Plk1 binding. Mutation of threonine 557 to alanine abolished interaction with Plk1, whereas mutation of two nearby serine residues in the conserved C-terminus of GCP-WD (S565A and S573A) had no effect (Fig. 2D). These results suggest that GCP-WD contains a phosphorylation-dependent Plk1 docking site. Alternatively, serine 418 and threonine 557 could also indirectly affect association with Plk1.

EGFP-tagged fragment C300, transiently expressed and immunoprecipitated from nocodazole-treated Hela cells, was phosphorylated *in vitro* after incubation with radioactively labeled ATP (Fig. 2E). Inhibition of Plk1 completely abolished phosphorylation, suggesting that coprecipitated endogenous Plk1 was responsible for this activity. The S418A and T557A mutants were not efficiently phosphorylated (Fig. 2E). Full length EGFP-tagged GCP-WD and the T557A mutant were expressed in cells and their phosphorylation state in mitotic extracts analyzed by western blotting. Compared to the wild type protein, the T557A mutant showed a slightly reduced mobility in high resolution SDS-PAGE, similar to the shift in mobility seen for endogenous GCP-WD after Plk1 inhibition (compare Fig. 2F with Fig. 2A). Plk1 inhibition did not further reduce the mobility of the T557A mutant (Fig. 2F). Together these results suggest that phosphorylation of GCP-WD at serine 418 is required for subsequent phosphorylation at other sites, including threonine 557. This promotes association with Plk1 and additional phosphorylation of GCP-WD.

### Mutation of the Plk1 binding and phosphorylation sites in GCP-WD does not affect centrosome targeting of GCP-WD

**Figure 3:**
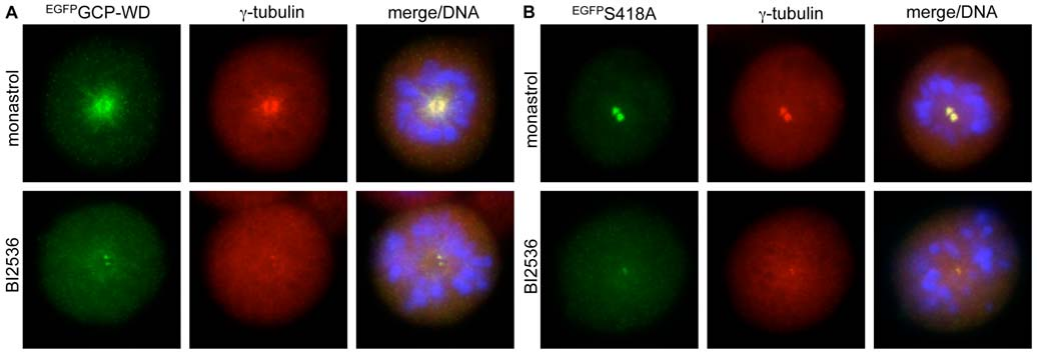
The S418A mutation does not affect centrosome localization of GCP-WD. HeLa cells depleted of endogenous GCP-WD and transiently expressing RNAi-resistant EGFP-tagged GCP-WD wt (A) or S418A mutant (B) were treated with monastrol or BI2536, fixed and immunostained with antibodies against GFP and γ-tubulin. DNA was visualised using DAPI.

Plk1 might control γ-tubulin targeting to mitotic centrosomes through phosphorylation of GCP-WD. One possibility is that phosphorylation of GCP-WD by Plk1 controls its interaction with the γTuRC. However, sucrose gradient analysis of interphase and mitotic cell extract as well as phosphatase-treated extract indicated that cofractionation of GCP-WD with the γTuRC is independent of its phosphorylation state (data not shown). Alternatively, phosphorylation of GCP-WD by Plk1 might regulate its interaction with the centrosome. To test this hypothesis we constructed RNAi-resistant, EGFP-tagged GCP-WD and a series of mutants carrying alanine mutations in either Plk1 binding (S418A, T557A) or Plk1 consensus phosphorylation motifs (S433A, T487A, S433A/T487A double mutant). These proteins were expressed in cells depleted of endogenous GCP-WD by RNAi. With the exception of the S418A mutant, all other mutants localized to centrosomes and to spindle microtubules, and supported the targeting of γ-tubulin to these structures, similar to the wild type protein. In addition, cells expressing these mutants had no obvious defect in bipolar spindle formation and chromosome segregation (data not shown). The S418A mutation interfered with spindle targeting but did not cause any defect in the accumulation of GCP-WD and γ-tubulin at mitotic centrosomes, confirming our previous results [13] (Fig. 3). Moreover, the centrosome accumulation of both wild type and S418A mutant EGFP fusion proteins was responsive to Plk1 inhibition (Fig. 3A,B), as observed for endogenous GCP-WD (Fig. 1). Plk1 inhibition also prevented localization of tagged wild type GCP-WD to spindle microtubules (Fig. 3A). These findings are consistent with the interpretation that Plk1 only indirectly regulates centrosome accumulation of GCP-WD rather than by direct binding and phosphorylation.

### Plk1 inhibition abolishes centrosomal accumulation of several proteins involved in γ-tubulin recruitment

**Figure 4:**
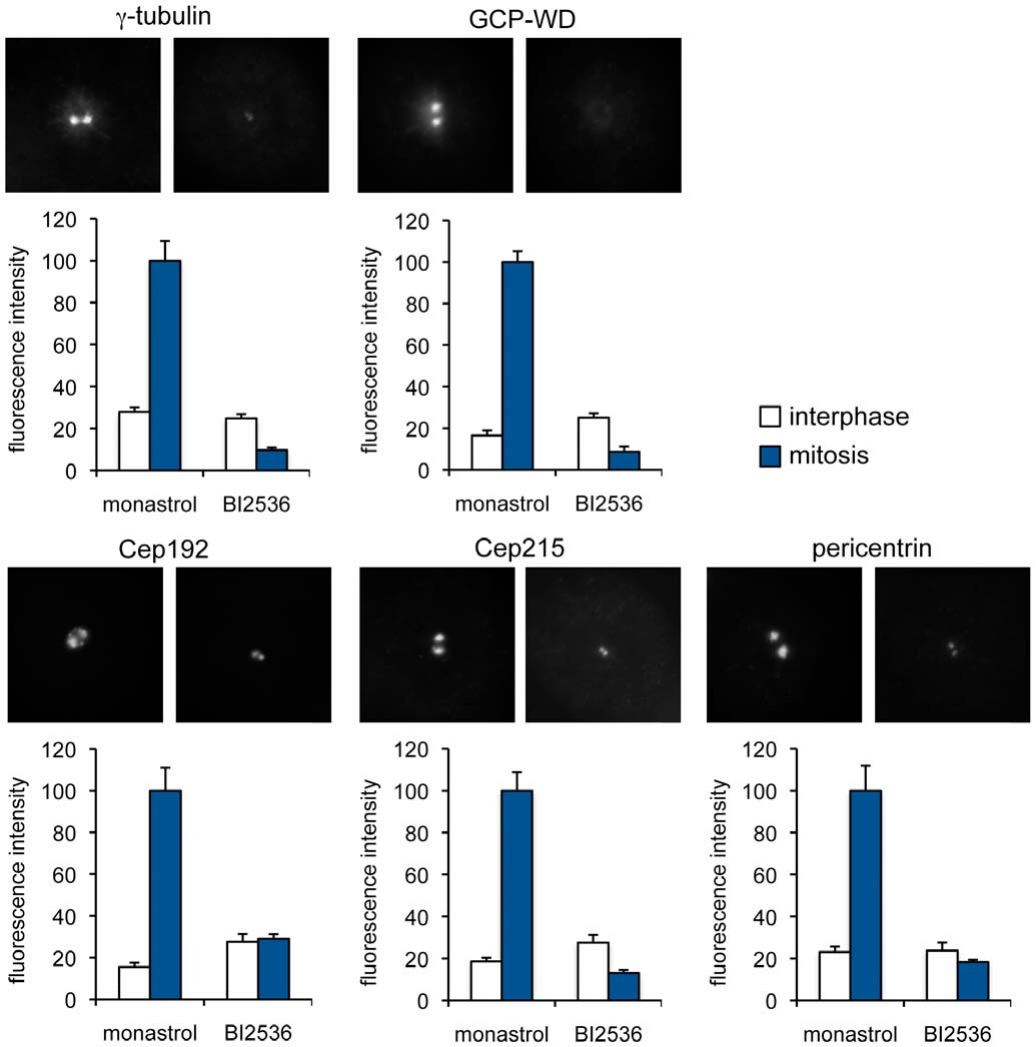
Plk1 inhibition interferes with the recruitment of Cep192, Cep215 and pericentrin to mitotic centrosomes. HeLa cells were treated with monastrol or BI2536, fixed and immunostained for GCP-WD, γ-tubulin, Cep192, Cep215 or pericentrin. Fluorescence intensities at the centrosomes were quantified in interphase and in mitosis (n≥10 cells/20 centrosomes per condition). Intensities were plotted as a percentage of the intensities measured in mitotic cells treated with monastrol. Error bars: SEM, 95% CI.

We tested whether Plk1-dependent γ-tubulin recruitment during centrosome maturation might involve PCM components other than GCP-WD. Cep192/hSPD2 [24], [25], pericentrin [24], [26] and Cep215/Cdk5Rap2 [27] have previously been implicated in γ-tubulin recruitment to centrosomes in mitosis. However, whether the centrosomal localization of these proteins is also regulated by Plk1 was not investigated. Strikingly, Plk1 inhibition impaired the accumulation at mitotic centrosomes of all these proteins as demonstrated by staining with specific antibodies, whereas localization to interphase centrosomes was not reduced (Fig. 4). Quantification of the signals revealed that centrosomal Cep192 and pericentrin in mitotic cells were reduced to interphase levels, whereas the centrosomal signal of Cep215 dropped, similar to that of GCP-WD and γ-tubulin, even below the signal at interphase centrosomes.

**Figure 5:**
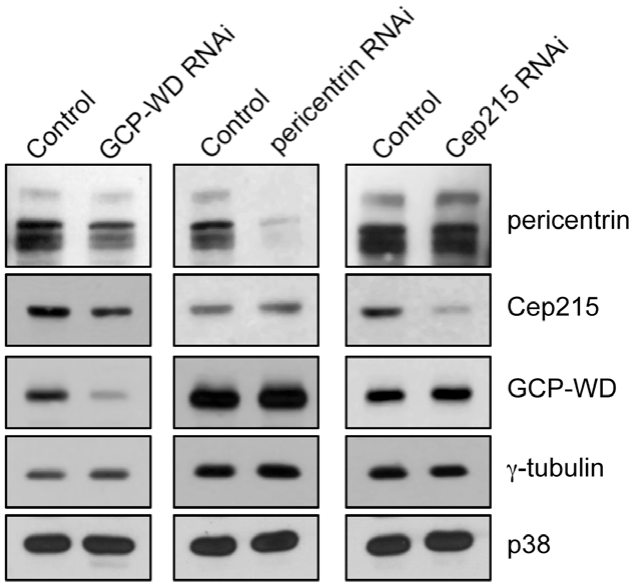
RNAi-mediated depletion of GCP-WD, pericentrin and Cep215, respectively, does not affect the levels of other centrosome proteins. After RNAi-mediated depletion of the indicated proteins in Hela cells, whole cell extracts were analyzed by westernblotting using specific antibodies as indicated. The p38 protein was used as a loading control.

**Figure 6:**
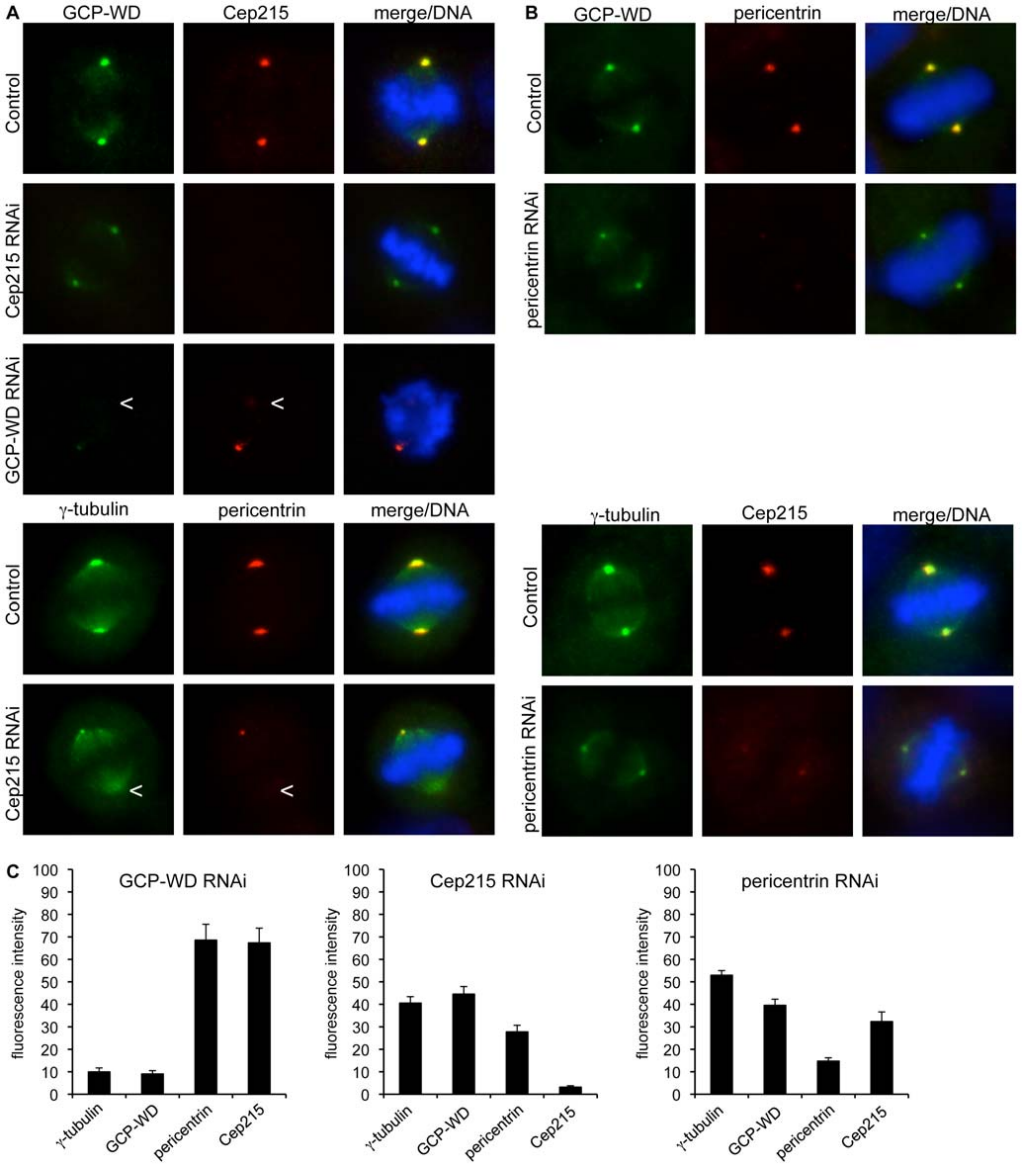
Cep215 and pericentrin depend on each other for localizing to mitotic centrosomes and function upstream of GCP-WD in γ-tubulin recruitment. (A) HeLa cells transfected with plasmids expressing shRNA targeting Cep215 or GCP-WD, were fixed and stained for GCP-WD and Cep215 or γ-tubulin and pericentrin. DAPI was used to label DNA. As a control cells were transfected with empty vector. In some panels one of the two centrosomes is out of focus and only one centrosome can be seen. The arrowhead marks the position of the out-of-focus centrosome, which in both cases was labelled with similar intensity as the one shown. (B) HeLa cells transfected with siRNA targeting pericentrin were processed for immunofluorescence with antibodies against γ-tubulin and Cep215 or GCP-WD and pericentrin. DAPI was used to visualize DNA. Unspecific RNA was transfected as a control. (C) After depletion of the indicated proteins by RNAi cells were fixed and stained with antibodies against γ-tubulin, GCP-WD, pericentrin and Cep215. Fluorescence intensities at mitotic centrosomes were quantified (n≥10 cells/20 centrosomes per condition). Intensities were plotted as a percentage of the intensities measured in control cells. Error bars: SEM, 95% CI.

Cep192 has recently been shown to function upstream of GCP-WD in the recruitment of γ-tubulin to mitotic centrosomes [24], [25], whereas the roles of pericentrin and Cep215 are less clear. We addressed this issue by systematic testing of interdependencies between Cep215, pericentrin and GCP-WD for their localization to mitotic centrosomes using RNAi. Western blotting of whole cell extract after RNAi confirmed that each protein was efficiently depleted without affecting the levels of the other proteins (Fig. 5). Cep215 depletion resulted in a reduction of both GCP-WD and γ-tubulin (∼60% reduction, respectively; Fig. 6A and 6C). This reduction was moderate considering the almost complete depletion of centrosomal Cep215 in these cells and was less severe than after treatment with Plk1 inhibitor (>90% reduction; Fig. 4). In Cep215 depleted cells centrosomal pericentrin was strongly reduced (Fig. 6A and 6C) and pericentrin depletion in turn also inhibited centrosome localization of Cep215 (Fig. 6B and 6C). RNAi of pericentrin also resulted in a decrease in the amount of centrosomal γ-tubulin as described [24], [26] and of centrosomal GCP-WD (Fig. 6B and 6C). However, pericentrin depletion caused only a partial loss of γ-tubulin and GCP-WD from the mitotic centrosomes, similar to that caused by Cep215 depletion (50–60% reduction). Importantly, depletion of GCP-WD, while resulting in efficient loss of centrosomal γ-tubulin as described, had no strong effect on the amount of centrosomal pericentrin [13], [14] and Cep215 (∼30% reduction, respectively; Fig. 6A and 6C). These results indicate a strong interdependency between pericentrin and Cep215 for their localization to mitotic centrosomes and that either protein only partially contributes to γ-tubulin recruitment. In contrast, GCP-WD is absolutely required for γ-tubulin recruitment and seems to function more proximal to the γTuRC in the recruitment pathway, most likely downstream of Cep215/pericentrin.

## Discussion

In addition to its function in spindle formation and cytokinesis Plk1 has a well established role in centrosome maturation at the G2/M transition, which involves the recruitment of additional γTuRCs from the surrounding cytoplasm. A longstanding goal in the analysis of this pathway has been the identification of a Plk1 substrate that regulates this process. Our results suggest that Plk1 regulates γ-tubulin recruitment to mitotic centrosomes by controlling the centrosomal levels of the γ-tubulin targeting factor GCP-WD, and that GCP-WD itself is a Plk1 substrate. However, by analyzing GCP-WD mutations that disrupt interaction with Plk1 (S418A, T557A) and mutations in potential Plk1 phosphorylation sites (S433A, T487A) we find no evidence for defects in centrosome targeting.

What is the role Plk1 in the regulation of GCP-WD? One possibility is that Plk1 regulates the interaction of GCP-WD with the mitotic spindle, which depends on phosphorylation of serine S418 and is critical for spindle-targeting of the γTuRC and the formation of additional microtubules within the spindle [13]. Might Plk1 be responsible for serine 418 phosphorylation? We consider this unlikely because serine 418 does not correspond to a Plk1 consensus phosphorylation site, and because the S418A mutation strongly reduces the global phosphorylation of GCP-WD, whereas Plk1 inhibition has only mild effects (as judged by changes in GCP-WD mobility in SDS-PAGE). However, serine 418 phosphorylation could promote additional phosphorylation by Plk1 at a different site, which could contribute to the interaction of GCP-WD with the spindle. This is supported by our observation that Plk1 inhibition not only prevents accumulation of GCP-WD and γ-tubulin at centrosomes, but also reduces their spindle localization. This would impair microtubule formation within the spindle and could contribute to the defects in kinetochore fiber assembly after Plk1 inhibition [8], [9]. However, our results for the T557A mutant, which is defective in Plk1-binding and phosphorylation but does not show any localization defects, suggest that regulation of GCP-WD by Plk1 is not essential for spindle localization of γTuRCs. Instead, Plk1 might regulate the augmin complex, which mediates spindle interaction of GCP-WD/γTuRC without affecting centrosome targeting [15], [16]. Interestingly, FAM29A/hDgt6, a subunit of the human augmin complex, was recently identified as a Plk1 substrate [16].

**Figure 7:**
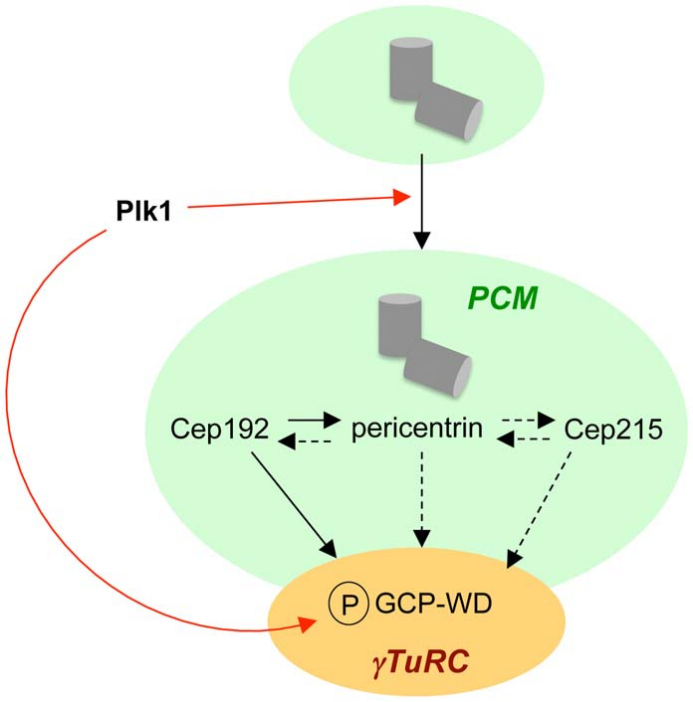
Interdependencies of components involved in the recruitment of γ-tubulin complexes to mitotic centrosomes. During centrosome maturation Plk1 phosphorylates PCM components, which results in the accumulation of PCM including γTuRC recruitment factors at mitotic centrosomes. Plk1-dependent phosphorylation of GCP-WD does not affect centrosome recruitment of γTuRCs, which is regulated upstream of GCP-WD. Upstream regulators and potential Plk1 substrates include Cep192, pericentrin, and Cep215. Pericentrin and Cep215 contribute to each other's centrosome localization and to γTuRC binding. Pericentrin is also important for centrosome localization of Cep192, which has the strongest impact on centrosome recruitment of GCP-WD/γTuRC [24], [25]. Solid black arrows indicate an essential role in the recruitment of the component they are pointing at, dashed black arrows indicate a partial role. Red arrows indicate Plk1-dependent regulation.

Our data show that in addition to GCP-WD several other centrosome proteins with a known role in γTuRC recruitment are also mislocalized after inhibition of Plk1. One of these proteins, Cep192, has recently been shown to function upstream of GCP-WD in centrosome targeting of γ-tubulin [24], [25]. Cep192 is also required for the recruitment of pericentrin to mitotic centrosomes. These results indicate that at least one Plk1-dependent step in γTuRC recruitment takes place upstream of GCP-WD and might promote the recruitment of other γTuRC attachment factors. Several lines of evidence suggest that γTuRC recruitment to mitotic centrosomes might not follow a simple pathway based on a single adaptor protein, but rather be the result of complex interactions between several PCM proteins. For example, pericentrin has been suggested to directly interact with γTuRC subunits and recruit γTuRCs to mitotic centrosomes [26], [28], [29], but compared to Cep192 the depletion of pericentrin causes only a moderate loss of centrosomal γ-tubulin [24](this study). However, there is also some mutual dependency between Cep192 and pericentrin for their localization to mitotic centrosomes, which complicates the interpretation of depletion phenotypes [24], [25]. Cep215/Cdk5Rap2 is yet another protein suggested to function in the recruitment of γTuRCs to centrosomes [27]. Similar to the homologous proteins Mto1 in yeast and Cnn in flies [30], [31], Cep215 was shown to interact with γ-tubulin complexes, but its relationship with GCP-WD was not investigated. Cep215 also associates with pericentrin to promote PCM cohesion at interphase centrosomes [32]. We have shown that there is interdependency between pericentrin and Cep215 for their localization to centrosomes in mitosis and depletion of either protein causes a similar partial loss of both GCP-WD and γ-tubulin from mitotic centrosomes. GCP-WD depletion only weakly affects centrosomal accumulation of these proteins and this effect might be the result of PCM fragmentation after mitotic arrest of GCP-WD depleted cells [13], [14], [25] rather than a direct effect on Cep215/pericentrin. We propose to place pericentrin and Cep215 together with Cep192 upstream of GCP-WD in the recruitment pathway (Fig. 7).

Our finding that Cep215 depletion results in a much weaker effect than Plk1 depletion or inhibition contrasts with data from a recent genome-wide RNAi screen in flies. Plk1 and the Cep215 homolog Cnn were identified as the genes with the strongest impact on centrosome maturation and Cnn was shown to be phosphorylated in a Plk1-dependent manner [33]. Most importantly, however, homologs of GCP-WD, Cep192, pericentrin were also identified as components involved in centrosome maturation [33]. In addition, myomegalin, another mammalian protein with homology to Cnn, might participate in centrosome maturation in the absence of Cep215[34].

It is important to note that the recruitment of γ-tubulin to mitotic centrosomes might be mechanistically different from its interaction with interphase centrosomes. In interphase γTuRC binding to centrosomes involves Ninein-like protein (Nlp). However, at the onset of mitosis, Plk1 phosphorylates Nlp and abolishes its interaction with centrosomes and with the γTuRC [35]–[37]. This might clear the way for the involvement of mitosis-specific recruitment factors.

To understand the complex interplay of different PCM proteins in mitosis and their impact on centrosome maturation in mammalian cells it will be important to test proteins such as Cep192, Cep215, and pericentrin for mitotic phosphorylation and determine the phosphorylation sites. Phosphorylation mutant analysis will reveal whether there is indeed one key substrate that initiates centrosome maturation or, alternatively, Plk1-dependent phosphorylation of several proteins, acting in parallel or complementary pathways, is required for efficient centrosome maturation and accumulation of γTuRCs.

## Materials and Methods

### Cloning and RNA oligonucleotides

A plasmid expressing Myc-tagged Plk1 was kindly provided by Erich Nigg, Martinsried, Germany. The expression construct for the EGFP-tagged C300 fragment of GCP-WD was generated by insertion into pEGFP-C1 (Clontech). A plasmid expressing full length GCP-WD-EGFP was constructed by inserting the GCP-WD sequence into pEGFP-N1 (Clontech). For simultaneous expression of shRNA from the same plasmid the GCP-WD shRNA expression cassette from pSUPER-GCP-WD [13] was excised (EcoRI/KpnI), blunted and inserted into the DraIII site of pGCP-WD-EGFP. Point mutations in the GCP-WD sequence to generate an RNAi resistant version [13] and other mutations were introduced by site-directed mutagenesis using the Quickchange protocol (Stratagene).

For RNAi experiments RNA oligonucleotides or pSUPER plasmids expressing shRNA were designed. The following sequences were used as targets: GCAGACATGTGTCAATTTA (GCP-WD), CAGCAGAACTGCTATTTAA (Cep215), GAGGACTTGGAACAGCTGCAGCAGA (pericentrin). For the depletion of Plk1 smartpool siRNAs (Dharmacon) were used.

### Cell culture and transfection

HeLa and U2OS cell lines were grown at 37°C in DMEM containing 10% fetal calf serum. Treatments with the Plk1 inhibitor BI2536 and with monastrol were done overnight at 100 nM and 100 µM, respectively. Nocodazole was used at 0.1 µg/ml to arrest cells in mitosis.

HeLa cells were transfected with plasmid or siRNA using Lipofectamine 2000 (Invitrogen). Depending on the target cells were analyzed 24–72 hours after transfection. For depletion of pericentrin, two consecutive transfections were performed as described [24]. U2OS cells were transfected using Fugene (Roche). For complementation of GCP-WD-depleted cells with exogenous GCP-WD and GCP-WD mutants we expressed from a single plasmid shRNA targeting endogenous GCP-WD and RNAi-insensitive EGFP-tagged GCP-WD or GCP-WD mutants. Cells were blocked in 2 mM thymidine 24 hours after the second of two consecutive transfections. After 20 hours cells were released and fixed 10 hours later.

### Antibodies and reagents

The following antibodies were used: mouse anti-Myc (monoclonal 9E10), rabbit anti-Myc (c-Myc A14; Santa Cruz), mouse anti-γ-tubulin (GTU-88, Sigma; TU-30, EXBIO/Axxora), mouse anti-α-tubulin (DM1α, Sigma), rabbit anti-GCP-WD [13], [14], mouse anti-GCP-WD (7D10, Abnova), rabbit anti-pericentrin [13], mouse anti-GFP (3E6, Molecular Probes), mouse anti-Plk1 (clone 36–206 for IP, clones PL2/PL6 for WB, Invitrogen), rabbit anti-Cep192 (kind gift of Laurence Pelletier, Toronto, Canada), mouse anti-HA (HA-7, Sigma), rabbit anti-Cdk5Rap2 (IHC-00063, Bethyl Laboratories).

All secondary antibodies used in immunofluorescence microscopy were from Molecular Probes, and peroxidase-coupled secondary antibodies used for western blotting were from Jackson Immunoresearch Laboratories. BI2536 for initial experiments was a generous gift from Aaron Straight, Stanford. For other experiments BI2536 and monastrol were kindly provided by the “Centre de Recherche en Oncologie Pierre Fabre”, nocodazole was from Sigma.

### High resolution SDS-PAGE

Extracts were prepared by shake-off of nocodazole-arrested U2OS cells, after wash in PBS, and resuspension in lysis buffer (50 mM Tris pH 7.5, 150 mM NaCl, 2 mM EDTA, 0.5% Triton X-100, 1 mM DTT, with a protease inhibitor mix (Sigma-Aldrich)). Extracts were sonicated and subjected to SDS-PAGE using 20 cm gels containing 6 or 7.5% acrylamide:bis-acrylamide at a ratio of 100∶1.

### Immunoprecipitations

Mitotic extracts were prepared from nocodazole-arrested cells after shake-off. Cells were washed in PBS and lysed (50 mM Hepes pH 7.4, 100 mM NaCl, 1 mM MgCl2, 1 mM EDTA, containing proteases and phosphatases inhibitors) for 30 min on ice. Lysates were prepared by by passing the cell suspension repeatedly through a small needle and centrifugation for 15 min at 13000 rpm at 4°C. Pre-cleared supernatants were incubated with antibodies for 2 hours at 4°C. Protein G (Plk1 antibody) or protein A (GCP-WD antibody) beads were added for an over night incubation. Beads were washed twice in lysis buffer + NP40 0.1%, then in 50 mM Hepes pH 7.4, 100 mM NaCl + NP40 0.1%, and finally in 50 mM Hepes pH 7.4 + NP40 0.1%.

For immunoprecipitation of Myc- and EGFP-tagged proteins transfected Hela cells were washed in PBS and lysed (50 mM HEPES, pH 7.5, 150 mM NaCl, 1 mM MgCl_2_, 1 mM EGTA, containing protease inhibitors and 0.5% TX100) for 5 min on ice. For the *in vitro* phosphorylation assay cells were treated with nocodazole for 5 hours before harvesting to enrich mitotic cells. After centrifugation for 10 min at 16,000 *g* at 4°C cleared lysates were incubated with antibodies for 1 h at 4°C. Protein G beads were added and the mixture was incubated for an additional hour at 4°C. The beads were pelleted and washed five times with lysis buffer + 0.5% TX-100.

For phosphatase treatment 0.5 µl λ-phosphatase (NEB) was added followed by incubation at RT for 15 min.

For *in vitro* phosphorylation the immunoprecipitates were incubated for 30 min at 30°C with 200 uM [γ-32P]ATP in kinase buffer (20 mM Hepes, pH 7.5, 50 mM NaCl, 5 mM MgCl2, 1 mM EGTA, 1 mM DTT). To inhibit Plk1 activity 10 nM BI2536 was added.

Samples were prepared for SDS-PAGE by boiling in sample buffer.

### Fluorescence microscopy

HeLa cells grown on coverslips were fixed in methanol at −20°C and processed for immunofluorescence following standard protocols. Images were acquired on a Zeiss Axiovert 200M microscope using Axiovision software (Carl Zeiss Microimaging, Inc.) or on a Leica DMI6000B microscope using AF6000 software (Leica). Fluorescence intensities at centrosomes were quantified on images acquired under constant exposure for each antigen. For quantification signal intensities in a circular area of 2 µm diameter surrounding each centrosome were measured using Axiovision, AF6000 and ImageJ software. An adjacent area of the same dimensions within each cell was quantified and subtracted as background.
